# Comparing emotional working memory in adolescents and young adults with and without depressive symptoms: developmental and psychopathological differences

**DOI:** 10.1186/s40359-022-00836-2

**Published:** 2022-05-25

**Authors:** Estíbaliz Royuela-Colomer, Laura Wante, Izaskun Orue, Caroline Braet, Sven C. Mueller

**Affiliations:** 1 Department of Personality, Psychological Assessment and Treatment, University of Deusto, Bilbao, Spain; 2 Department of Developmental, Personality, and Social Psychology, Ghent University, Ghent, Belgium; 3 Department of Experimental Clinical and Health Psychology, Ghent University, Ghent, Belgium

**Keywords:** Affective processing, Development, Depression, N-back, Working memory

## Abstract

Depressive symptoms are associated with working memory impairments. Yet, comparative studies examining working memory across the developmental spectrum in depressed and non depressed cohorts are lacking. This study examined emotional working memory in 74 adolescents (mean age = 14; 21 with depressive symptoms) and 92 adults (mean age = 22; 36 with depressive symptoms). Participants completed two versions of an emotional face n-back task, and either paid attention to the valence of the emotion or the gender. Both tasks were completed at low load (0-back) and high load (2-back). In the high load condition, healthy adolescents showed a bias towards positive faces, both speeding up reaction times (RTs) when emotion was task relevant but slowing RTs when they were task irrelevant. This interaction was neither significant in adolescents with depressive symptoms nor in young adults. Depressive symptoms did not influence RTs in low load. The results indicate that adolescents with depressive symptoms might lack the bias towards positive affective material at high load WM task present in healthy adolescents.

**Supplementary Information:**

The online version contains supplementary material available at 10.1186/s40359-022-00836-2.

## Background

Depressive symptoms are common at any age, with prevalence rates of major depressive disorder of around 3% in children, 5–20% in adolescents, and 7–13% in adults [1–4]; with a high correlation among them [5]. Diagnostic criteria of DSM-5 [6] and ICD-11 [7] are almost identical for adolescents and adults, although an important affective–cognitive difference in DSM-5 acknowledges that adolescents experience irritable rather than sad mood. However, several studies also documented etiological, symptom profile, and treatment differences between adolescent and adult depression [8–10], raising questions regarding the similarity of depression across age groups. For example, Rice et al. [8] observed that anhedonia, loss of interest, and concentration problems were common among adults, whereas, in adolescents, appetite and weight changes, loss of energy, and insomnia prevailed.

When considering mental health problems, one crucial cognitive risk factor constitutes deficits in cognitive control and working memory (WM) specifically [11, 12]. In particular, a growing body of literature has examined how affective material influences WM. For example, meta-analytic evidence concluded that affective information modulates WM performance, as seen in behavioral and neuroimaging studies [13, 14], and this is mainly the case for individuals suffering from mental health problems [14]. As Schweizer and colleagues indicated in their meta-analysis [14], response times for affective stimuli, compared to neutral stimuli, were slower on behavioral measures of WM, including simple spam tasks, n-back tasks, delayed-match-to-sample tasks, and complex spam tasks. Of the 165 studies reviewed by Schweizer, the n-back task was the most frequently used; 63 studies looked at the n-back task, 44 of those used both positive and negative stimuli. However, only two of these studies included depressed participants, whilst none of these studies compared adolescents and adults.

Several studies found an impairment in WM tasks for negative material—when compared to positive or neutral material—among depressed adults [15–18]. By contrast, results from adolescents are mixed [19–21]. Whereas Tavitian et al. [21] documented impaired WM by neutral irrelevant information but not by positive or negative information in depressed versus healthy comparison adolescents, Ladouceur et al. [20] found that irrelevant negative information impaired WM performance in a clinical sample of early adolescents.

Some of these prior discrepancies may stem from the type of negative emotion involved. Whereas some studies used ‘sadness’ [18], an emotion congruent with depression, other studies used anger [19, 21]. In contrast to sadness, anger is not emotionally congruent but has also been documented as a potent trigger of negative emotionality [22]. These studies highlight the importance of studying the differential impact of distinct or specific affective stimuli, both relevant and irrelevant, and acknowledge including depressed and healthy comparisons. However, direct comparisons across age and psychopathology are presently lacking.

Arguably, because WM is not fully mature until the age of 19 [23] and as emotion appears to influence WM differently in healthy adults versus adolescents [24]—with stronger working memory effects for adolescents when compared to adults—one might anticipate a differential age effect in individuals with depressive symptoms. Strikingly, studies directly comparing WM and cognitive control in adolescents and adults with and without depressive symptoms together with two-matched control groups are presently non-existent in the literature. Whereas one study compared adolescents and adults but only had a clinical depression group for adolescents [25], another study examined the association between affective control, mental health difficulties, and age group (early and mid- adolescents and adults), but did not include any symptomatic comparison group [26].

Therefore, to explore developmental and psychopathology differences in WM performance, this study tested the influence of relevant and irrelevant affective stimuli in adolescents and young adults with and without depressive symptoms, using a well-validated emotional WM task [19, 24, 27]. As multiple studies suggest that depressed adults experience greater difficulty in manipulating material in WM compared to healthy comparisons [16], especially when the material is negative [15, 17, 18], we hypothesized that when exposed to anger, this would have a bigger influence on WM in those with depressive symptoms. Because adolescents have a heightened reactivity to emotional content [24, 28, 29], we hypothesized that the influence of affective material would be stronger in adolescents—both healthy and with depressive symptoms—compared with young adults. Adolescents’ data were used from the study of Wante et al. [19]; for the purpose of this study and to extend Wante’s results, two groups of adults (healthy and with depressive symptoms) were added.

## Method

### Participants

In total, 166 participants completed the study (60 male; 34.3% with depressive symptoms). Of those, 74 were adolescents (21 with depressive symptoms, mean age = 14.76 years, *SD* = 1.64; 53 healthy, mean age = 14.27 years, *SD* = 1.45) and 92 young adults (36 with depressive symptoms, mean age = 21.69 years, *SD* = 2.83; 53 healthy, mean age = 21.79 years, *SD* = 3.79) (Table 1). Adolescents’ data were used from the study of Wante et al. [19], but data from the adults have not been published before.

**Table 1:** Demographic characteristics of participants

	Adolescents			Young adults		
	Total	Depressive symptoms	Healthy	Total	Depressive symptoms	Healthy
N	74	21	53	92	36	56
Age (SD)	14.41 (1.51)	14.76 (1.64)	14.27 (1.45)	21.75 (3.43)	21.69 (2.83)	21.79 (3.79)
Gender, N (%)						
Female Male	48 (64.9) 26 (35.1)	19 (90.5) 2 (9.5)	29 (54.7) 24 (45.3)	58 (63) 34 (37)	25 (69.4) 11 (30.6)	33 (58.9) 23 (41.1)
Depression						
Mean (SD)	11.04 (9.3)	23.74 (8.5)	6.4 (3.3)	13.65 (12.2)	26.06 (10.1)	5.68 (4.2)
Anxiety						
Mean (SD)	35.85 (8.9)	46.7 (5.6)	31.96 (6)	44.46 (12.7)	45.85 (13.2)	43.57 (12.4)

For depression, CDI (adolescents), BDI (adults) scores are reported; Standard deviations are in parenthesis. For anxiety, STAI Trait-State scores are reported; Standard deviations are in parenthesis

In Wante et al. [19], a semi-structured clinical interview was used for classification, and depressed adolescents were also included in the clinical group even if their depression scores (based on the Children’s Depression Inventory; CDI [30]) were low but had a diagnosis of depression. Because the goal of this study was to use a common dimensional cutoff for both adolescents and adults, these individuals with a history of depression were excluded. In this present study, groups were allocated according to participants’ scores on the Beck Depression Inventory-II (BDI-II) [31] (young adults) and CDI [30] (adolescents). A growing body of literature supports the use of cut-off scores for these two instruments [32–34], and a considerable amount of literature has been conducted using depression as a categorical variable based on these exact BDI/CDI scores [35, 36]. This also resulted in slightly different sample sizes/grouping relative to the Wante et al. [19] study.

Inclusion criteria for adolescents were age between 10 and 18 years, and IQ within the normal range (> 70). All adolescents signed informed assent, and their legal guardians signed informed consent. Young adults were recruited through online screening at the faculty. The majority of participants were Belgian. After signing the informed consent, adult participants completed the BDI-II [31] to assess current depressive symptomatology. Inclusion criteria for this study were *set a priori* as a CDI [30] (adolescents) / BDI-II [31] (adult) score of 14 or above (depressive symptom group) or 13 or less (healthy group). A CDI/BDI-II score of ≥ 14 commonly denotes mild to more severe depression in the literature [30, 31]. As compensation, adolescents received two cinema tickets and adults 16 EUR. The ethical committee of the Faculty of Psychology and Educational Sciences at Ghent University approved the study.

### Measures

#### Emotional n-back task

The n-back task has previously been validated to compare healthy versus depressed youths [19] and healthy adolescents versus adults [24, 27], and was thus optimal for cross-age comparisons. The task was programmed in Presentation Software and was run on a 15.6-inch Dell laptop. Images were selected from the NimStim [37] and the Radboud Faces Databases [38]. Images included 32 adult actors (16 male and 16 female), and each actor posed the three emotional expressions (neutral, happy and angry), resulting in 96 pictures. Although exact arousal/valence ratings are not available for the NimStim dataset, low arousal versions (individuals expressing emotions with their mouths closed) were selected. Images were grayscaled, and the background hair of faces was removed using Adobe Photoshop 5.0. Following the procedures used in a previous study by Cromheeke et al. [24], images were displayed on a black background at 320 × 400 pixels, corresponding to approximately 8 × 10 cm. For the practice trials, 14 pictures of different actors were selected.

Participants completed a low cognitive load (0-back) and a high cognitive load (2-back) version of the *n-*back task. As it has been described in previous studies [19, 24], each version included two conditions, a *gender* condition, in which participants needed to focus on the gender of the faces, and a *valence* condition, in which participants needed to focus on the emotional expression of the faces. In the 0-back task, participants needed to respond to a target. In the gender condition, the target was a male or a female, whereas the target was the emotion in the valence condition. Participants were instructed to press the left mouse button if the presented face was the target and the right mouse button if the presented face was not a target. In the 2-back task, participants needed to compare the gender (in the gender condition) and the emotion (in the valence condition) of the current face with the face presented two trials before. The 2-back task consisted of match and mismatch trials. A match trial refers to a trial in which the gender or the emotional expression of the faces are the same, whereas a mismatch trial refers to a trial in which gender or the emotional expression of the faces are different. Participants were asked to press the left mouse button for a match trial and the right mouse button for a mismatch trial. Pictures were presented for 2000 ms, with a 500-ms inter-trial interval.

### Questionnaires

#### Depression inventory

The BDI-II [31] is a frequently used questionnaire assessing depressive symptomatology in adults according to the DSM-IV criteria [39]. The questionnaire assesses cognitive, affective, and somatic aspects of depression and is scored from 0 to 63. The scale has excellent reliability in the Dutch version [40]. Scores of 14 to 19 indicate mild depression; scores above 19 moderate to severe [31].

The CDI [30, 41] is a 27-item self-report questionnaire designed to assess depressive symptoms in youths and was built on the BDI. The CDI showed good psychometric properties [41].

#### Trait anxiety inventory

The Trait version of the State-Trait Anxiety Inventory (adults: STAI, [42]; adolescents: STAI-C, [43, 44]) measures the frequency and intensity of trait anxiety symptoms with 20 items and has shown to be valid and reliable in the Dutch translation [45].

#### Procedure

All participants completed the task in testing rooms at the faculty. After signing informed consent and filling out the questionnaires, participants received task instructions on the computer screen repeated orally by the experimenter. Participants first completed a training phase, including 10 trials of the 0-back task and 24 trials of the 2-back task and were able to ask for help or clarification. Only participants who had at least 60% accuracy in the training phase could continue the task.

#### Data analyses

We performed a repeated-measures analysis of covariance (rmANCOVA) with load (0-back or 2-back), task condition (gender or valence), and emotion (angry, happy, or neutral) as within-subject factors and depression status (depressive symptoms or healthy) and age group (young adults or adolescents) as between-subjects factors for the mean reaction time (RTs, correct trials only) and accuracy (percentage correct responses), separately. Because depressive symptoms often occur with anxiety [46] and share structural and brain alterations in circuits involving emotion regulation and cognitive control [47], anxiety symptoms were introduced as a covariate. To follow up significant interactions, paired-samples *t-*tests were conducted with the Bonferroni-Holm correction for multiple comparisons. Effect sizes are reported as Cohen’s *d* or partial eta squared, as appropriate. Lower-order interactions were not considered if these were qualified by higher-order interactions comprising the same factors (but are provided in the Additional file 1: Supplementary material for the sake of completeness and transparency).

## Results

### Reaction time (RT)

**Fig. 1:**
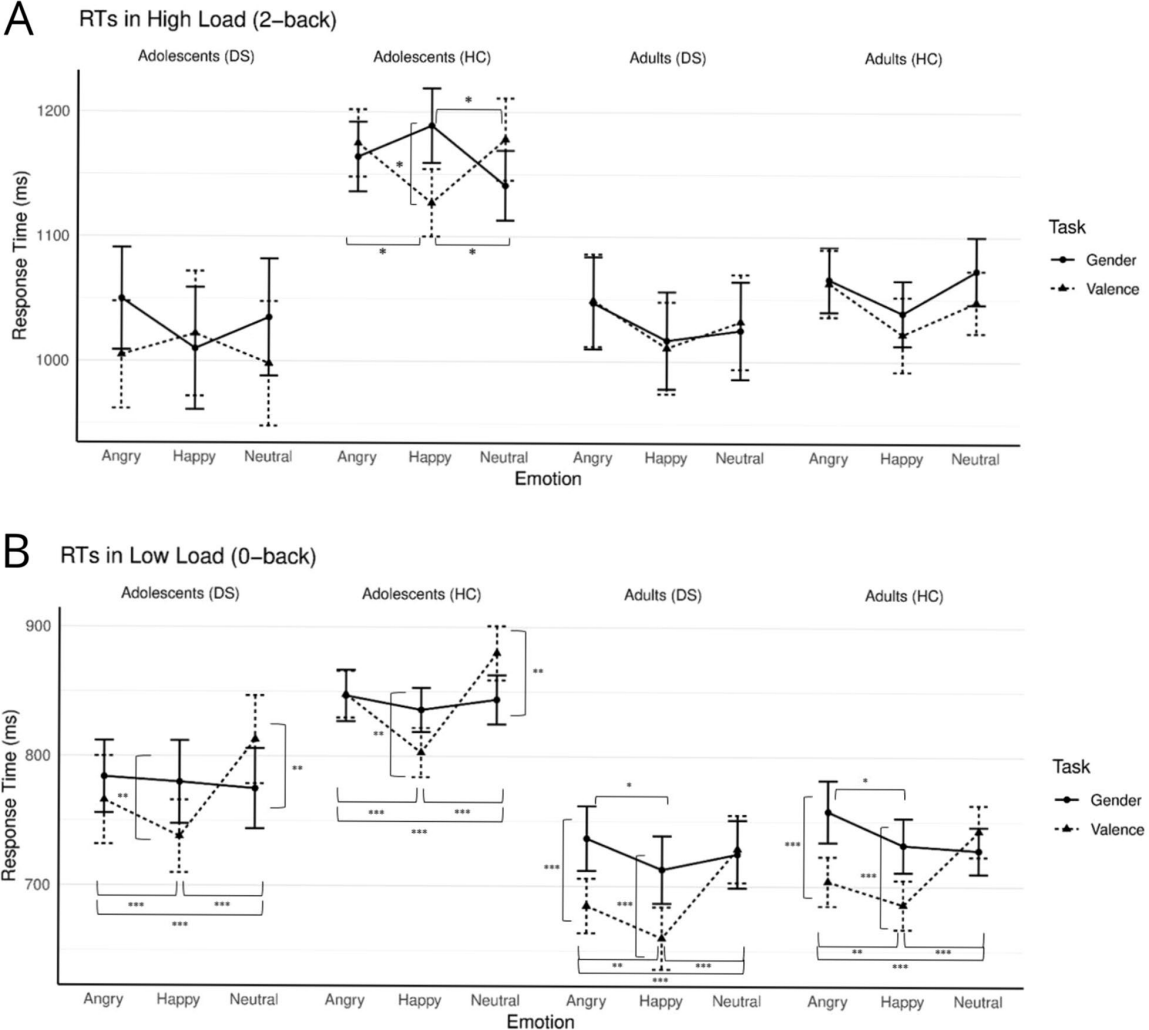
** A** Reaction times for gender and valence tasks of angry, happy neutral faces in high load task. **B** Reaction times for gender and valence tasks of angry, happy neutral faces in low load task. Error bars represent standard errors of the mean. *p < .05. **p < .01. ***p < .001. DS = Depressive Symptoms; HC = Healthy Comparisons

As predicted, a significant five-way interaction among load (0-back or 2-back), task condition (emotion or gender), emotion (happy, angry or neutral), depression status (depressive symptoms or healthy), and age group (young adults or adolescents) was found (*F*(2, 310) = 3.63, *p* = .028, ηp² = 0.02) (Fig. 1; Additional file 1: Table S1). The covariate anxiety did not affect these results. To break down this complex interaction, we followed it up by running two four-way interactions splitting it at the age group level (i.e., running the rmANCOVA for young adults and adolescents separately). Below, the results of the rmANCOVA conducted separately for the adult and adolescent groups are discussed successively.

### Young adults

Although the four-way interaction was not significant, there was a significant three-way interaction among load, task, and emotion (*F*(2, 178) = 5.69, *p* = .004, ηp² = 0.06) (see next paragraph for discussion of this interaction). Psychopathology group did not interact, or had a main effect. A main effect of load indicated faster RTs in low load relative to high load (*F*(1, 89) = 306.93, *p* < .001, ηp² = 0.78), a main effect of task indicated faster RTs in the valence relative to the gender condition (*F*(1, 89) = 5.34, *p* = .023, ηp² = 0.06) and a main effect of emotion (*F*(2, 178) = 18.10, *p* < .001, ηp² = 0.17) indicated faster RTs for happy than neutral, (*t*(91) = − 5.38, *p* < .001, *d* = 0.56) and happy than angry faces (*t*(91) = − 6.10, *p* < .001, *d* = 0.64). The covariate, anxiety, was significantly and negatively related to overall RTs (*F*(1, 89) = 6.34, p = .014, ηp² = 0.07) (i.e., higher anxiety = shorter RTs).

To examine the above three-way interaction a condition by emotion interaction was run for low load (0-back) and high load (2-back) separately. During high load, no significant interactions emerged (all *p*s > 0.05). There was a main effect of emotion (*F*(2, 180) = 8.66, *p* < .001, ηp² = 0.09), indicating faster RTs for happy than neutral, (*t*(91) = − 2.94, *p* = .008, *d* = 0.31) and happy than angry faces (*t*(91) = − 4.63, *p* < .001, *d* = 0.48), and a main effect of anxiety (*F*(1, 90) = 5.38, *p* = .023, ηp² = 0.06).

During low load (0-back) the two-way interaction of task condition by emotion was significant (*F*(2, 180) = 17.82, *p* < .001, ηp² = 0.17). RTs for angry and happy faces were faster when the emotion was relevant (valence condition) than when it was irrelevant (gender condition) (*t*(91) = 4.59, *p* < .001, *d* = 0.48 and *t*(91) = 4.53, *p* < .001, *d* = 0.47, respectively). Moreover, within the valence condition RTs were faster for happy compared to neutral (*t*(91) = − 6.41, *p* < .001, *d* = 0.67), happy compared to angry (*t*(91) = 2.78, *p* = .007, *d* = 0.29), and angry compared to neutral faces (*t*(91) = − 4.53, *p* < .001, *d =* 0.47). Within the gender condition, RTs for happy faces were faster than RTs for angry faces (*t*(91) = 2.96, *p* = .012, *d* = 0.31). There was also a main effect of task condition (*F*(1, 90) = 11.72, *p* = .001, ηp² = 0.12) — with faster RTs in the valence compared to the gender condition —and emotion (*F*(2, 180) = 12.84, *p* < .001, ηp² = 0.13); with faster RTs for happy compared to neutral (*t*(91) = − 4.96 *p* < .001, *d =* 0.52) and to angry (*t*(91) =− 4.00, *p* < .001, *d =* 0.42). The main effect of anxiety (*F*(1, 90) = 4.26, *p* = .042, ηp² = 0.06) indicated faster RT with higher anxiety.

### Adolescents

In adolescents, the four-way interaction was significant (*F*(2, 130) = 4.83, *p* = .009, ηp² = 0.07). Anxiety did not have any effect. A rmANCOVA with the task, emotion as within-participant factors, and psychopathology group as the between-participants factor was run separately for the low load (0-back) and high load (2-back) conditions.

In the high load condition, the three-way interaction among task, emotion, and psychopathology group was significant (*F*(2, 130) = 3.44, *p* = .035, ηp² = 0.05). To examine the three-way interaction a two-way rmANCOVA task condition by emotion was conducted separately for adolescents with and without depressive symptoms.

For adolescents with depressive symptoms, no significant findings emerged (all *p*s > 0.05). In healthy adolescents, the two-way interaction of emotion by task was significant, (*F*(2, 96) = 6.34, *p* = .003, ηp² = 0.12) indicating slower RTs for happy faces than neutral faces (*t*(52) = 3.01, *p* = .012, *d* = 0.41) in the gender condition but, faster RTs for happy faces relative to angry (*t*(52) = 2.92, *p* = .012, *d* = 0.40) and neutral faces (*t*(52) = − 2.98, *p* = .012, *d* = 0.41) during the valence condition, showing a performance benefit when happy faces were task-relevant. Moreover, RTs for happy faces were faster in the valence condition compared to the gender condition (*t*(52) = 2.41, *p* = .019, *d* = 0.33).

In the low load condition, the three-way interaction was not significant, and there was no main effect of psychopathology, but there was a significant two-way task condition by emotion interaction (*F*(2, 130) = 12.22, *p* < .001, ηp² = 0.16). For happy emotional faces, RTs were faster in the valence condition relative to the gender condition (*t*(73) = 2.91, *p* = .010, *d* = 0.34), whereas for neutral faces, RTs were faster in the gender condition relative to the valence condition (*t*(73) =− 3.50, *p* = .003, *d* = 0.41). For the gender condition, there were no significant differences on RTs between emotions. In the valence condition, there were significant differences in all the emotions; happy was faster than neutral (*t*(73) = − 7.83, p < .001, *d* = 0.91) and happy was faster than angry (*t*(73) = 4.48, *p* < .001, *d* = 0.52) and angry was faster than neutral (*t*(73) = − 3.62, *p* = .001, *d* = 0.42). There was also a main effect of emotion (*F*(2, 130) = 12.22, *p* < .001, ηp² = 0.16), *t* tests indicated that RTs for happy were faster than for anger (*t*(73) = − 4.71, *p* < .001, *d* = 0.55) and for neutral (*t*(73) = − 6.75, *p* <. 001, *d* = 0.78), and faster for angry compared to neutral faces (*t*(73) = − 2.32, *p* = .042, *d* = 0.27). Neither anxiety nor psychopathology revealed a main effect.

### Accuracy

In contrast to the findings in RTs, no significant five-way interaction emerged in accuracy (*p* = .291). Importantly, no main effects involving group were statistically significant, excluding any potential speed-accuracy trade-offs. Anxiety did not affect interactions, nor a main effect. However, there were 3 2-way interactions. The load by age group (*F*(1, 155) = 16.52, *p* < .001, ηp² = 0.10) interaction indicated higher accuracy for young adults when compared to adolescents (*t*(163) = − 7.01, *p* < .001, *d =* 1.10) during the high load condition, with no differences during the low load condition. Both groups showed fewer errors during low load as compared to high load (young adults: *t*(90) = − 11.55, *p* < .001; adolescents: *t*(73) = − 18.90, *p* < .001). Because the load by emotion and load by task interactions did not influence the main findings, it is not reported here but can be found in the Additional file 1: Supplementary material.

## Discussion

The main goal of the current study was to examine the differential effects of affective information on WM among young adults and adolescents with depressive symptoms and healthy comparisons relying on a standardized paradigm. Whereas Wante’s [19] prior study documented a lack of a positivity bias in depressed youths, the current experiment extends these prior findings by including two adult groups: with and without depressive symptoms. The most striking result is that, during high WM load, healthy adolescents showed a bias for positive emotions, improving (in valence condition) and impairing (in gender condition) performance, whereas this effect was not present in young adults or adolescents with depressive symptoms. Interestingly, there was also a general main effect of happy emotions, with faster RTs compared to neutral or angry emotions. Regarding the negative material, contrary to our expectations, angry faces did not affect RTs differently according to depressive symptoms. Concerning accuracy, we did not find a significant 5-way interaction but found that, only in high load, young adults, compared with adolescents, had fewer errors.

During low cognitive load, young adults showed shorter RTs (in the valence condition) and longer RTs (in the gender condition) to both happy and angry faces. By contrast, in adolescents, during low cognitive load, only RTs for happy faces were shorter in the valence condition relative to the gender condition, and there was no effect of angry faces. In line with our results, Cromheeke et al. [19] suggested that adolescents, compared to adults, are hypersensitive to positive stimuli, having their attention more easily captured by happy, but not for angry faces.

Contrary to previous studies suggesting that anxiety impairs WM performance [48], our results indicate that anxiety symptom severity did not influence the main results and had no effect in adolescents. However, there was a main effect of anxiety in young adults, which suggests that anxiety was negatively associated with overall RTs, but did not specifically impair WM performance, as its interaction with load, task or emotion was not significant.

The observed bias towards positive emotions in healthy adolescents during high WM load, is in line with the positive attenuation hypothesis [49]. According to the positive attenuation hypothesis [49], depressed individuals are characterized by reduced emotional reactivity to positive stimuli, such as insensitivity to positive information. The fact that adolescents, regardless of whether they had depressive symptoms or not, were sensitive to positive emotion in the low load could be attributed to the high cognitive effort needed to process information in high load, compared to low load, which could have been affected by depressive symptoms. The positivity bias in healthy adolescents is consistent with prior work among healthy adolescents, compared to clinical comparisons, in which positive affective material impaired performance—when used as a distractor [20]—, and increased performance—when used as the target stimuli [50]. The differential effect of cognitive load, on the other hand, can be explained by hypotheses that postulate that during demanding tasks, fewer cognitive resources will be available to process affective information [51, 52], possibly because all attentional resources are directed towards the target [53]. Consequently, since depression reduces cognitive resources [54, 55], there will be an emotional blunting effect in a high cognitive load scenario.

In comparison to adolescents, the positivity bias in young adults was absent, which supports recent research that documented a positivity bias in healthy adolescents, but not in healthy adults [27]. The age-specific modulations of working memory performance could be explained by neurodevelopmental differences in cognitive control emotion interactions. Specifically, one study found that happy faces elicited more nucleus accumbens activation in adolescents relative to adults, which might underlie the positivity bias we found in adolescents [27]. Moreover, our results might have been influenced by the developmental differences in WM [23, 56], and precisely, a heightened sensitivity to positive affective material in adolescents [50], which might explain why the positivity bias was not present in young adults during high load.

Interestingly, results from this study suggest that happy faces are generally detected more quickly than angry or neutral faces, which is in line with previous studies that found a happiness superiority effect [57, 58]. Since our sample comprised individuals with and without depressive symptoms, the fact that there was a main effect of happy faces, in general, could indicate that the effect of positive stimuli is independent of depression status. In addition, happy faces could have induced positive affect, which might have improved WM capacity, as a previous study indicated [59].

Contrary to expectations and earlier findings suggesting an attentional bias towards negative stimuli among individuals with depressive symptoms [15, 17, 60, 61], our results did not show an impact of negative stimuli during high cognitive load. However, some prior studies included affective and neutral words [15, 17] rather than pictures, as we did. Also, other previous work included sadness as the negative stimuli [17, 60, 61], whereas the current study relied on anger instead. Thus, attentional biases in depression might not be related to all negative stimuli but are specific to depression-related information (i.e., sad stimuli); this distinction was shown in earlier studies among adults [62, 63], and adolescents [64].

The lack of a negative affective bias is consistent with a recent review suggesting that contradictory findings on positive and negative affective bias might be explained by the difference in experimental paradigms, such as visual attention during zero back and working memory during 2 back [65]. An important factor influencing results pointed out in that review is stimulus choice/valence. Since neutral faces are more likely to be perceived as negative faces, an imbalance between positive and negative stimuli could have influenced the mixed results regarding angry faces. An alternative explanation could be that the impact of affective material in WM is minor at the performance level but more severe at the neural level [14]. For example, Harvey et al. found no differences between depressed and healthy participants in performance measures but differences at the neuronal level, suggesting that individuals with depression may need more cognitive resources to maintain the same performance as healthy individuals [66].

### Limitations

Despite the interesting findings, one limitation is the relatively small size of the adolescents with depressive symptoms. However, the sample size of adolescents with depressive symptoms is similar to previous works [16, 20], while the other three group’s sample size was reasonably large. Secondly, we do not know whether the findings would generalize to pre-adolescent children. However, finding appropriate tasks that are equally challenging to all age groups may be difficult. Third, although including angry faces as affective stimuli is seen as important and ecologically valid, future studies should include a broader range of affective stimuli before generalized conclusions can be made. In this vein, it is regrettable that more precise valence/arousal ratings were not available. However, given that the current effects emerged with low arousal versions (mouth closed), whether a future replication with high arousal images might find increased differences remains to be seen. Fourth, since IQ scores could influence WM tasks, including them in our study could have been interesting. However, since these were not collected for the adults, this has to be taken into consideration as a possible limitation. Of note, consistent with previously reported prevalence rates [2, 4], more females reported depression than males in the present sample. Although an analysis of gender differences on emotional WM may have been desirable, we do not think that analyses with our sample size would have yielded statistically reliable findings. Therefore, future studies should aim to design a study to address this issue directly.

## Conclusions

To our knowledge, this is the first study to present a full comparison between adolescents and adults with depressive symptoms and with two respective healthy comparison groups using the same cognitive task. The main finding suggests a bias towards positive affective material during high load WM in healthy adolescents. Since therapy for MDD is emotionally (and cognitively) demanding, treatments might benefit from cognitive control training to enhance and control emotional processing. For instance, recent research in this field points towards working memory training to regulate affective symptoms in adolescents [67, 68]. Our results suggest that intervention and prevention programs during adolescence might benefit from focusing on the processing of positive affective material. Furthermore, the findings might also relate to other therapeutic interventions in adolescents with depressive symptoms, such as mindfulness-based interventions, which effectively enhance positive emotion awareness [69, 70] and improve WM during adolescence [71]. However, further scrutiny of the findings to different therapeutic approaches is necessary.

## Supplementary Information

**Additional file 1**.** Supplementary Table 1**. Mean RTs (in Milliseconds) and Accuracy Rates (in %) on the 0-Back Task and the 2-Back Task for Young Adults and Adolescents as a Function of Psychopathology Group.

## Data Availability

The datasets used and/or analyzed during the current study are available from the corresponding author on reasonable request.

## References

[1] Lim GY, Tam WW, Lu Y, Ho CS, Zhang MW, Ho RC (2018). Prevalence of depression in the community from 30 countries between 1994 and 2014. Sci Rep.

[2] Mojtabai R, Olfson M, Han B. National trends in the prevalence and treatment of depression in adolescents and young adults. Pediatrics. 2016;138:Article e20161878. 10.1542/peds.2016-1878. 10.1542/peds.2016-1878 PMC5127071 27940701

[3] Kessler RC, Walters EE (1998). Epidemiology of DSM-III-R major depression and minor depression among adolescents and young adults in the national comorbidity survey. Depress Anxiety.

[4] Hankin BL, Young JF, Abela JRZ, Smolen A, Jenness JL, Gulley LD (2015). Depression from childhood into late adolescence: influence of gender, development, genetic susceptibility, and peer stress. J Abnorm Psychol.

[5] Johnson D, Dupuis G, Piche J, Clayborne Z, Colman I (2018). Adult mental health outcomes of adolescent depression: a systematic review. Depress Anxiety.

[6] American Psychiatric Association. Diagnostic and Statistical Manual of Mental Disorders. 5th edition. Washington, DC; 2013.

[7] World Health Organization. International statistical classification of diseases and related health problems. 11th edition. 2019.

[8] Rice F, Riglin L, Lomax T, Souter E, Potter R, Smith DJ (2019). Adolescent and adult differences in major depression symptom profiles. J Affect Disord.

[9] Weiss B, Garber J (2003). Developmental differences in the phenomenology of depression. Dev Psychopathol.

[10] Rohde P, Lewinsohn PM, Klein DN, Seeley JR, Gau JM (2013). Key characteristics of major depressive disorder occurring in childhood, adolescence, emerging adulthood, and adulthood. Clin Psychol Sci.

[11] Zhang D, Xie H, He Z, Wei Z, Gu R (2018). Impaired working memory updating for emotional stimuli in depressed patients. Front Behav Neurosci.

[12] Snyder HR (2013). Major depressive disorder is associated with broad impairments on neuropsychological measures of executive function: a meta-analysis and review. Psychol Bull.

[13] Cromheeke S, Mueller SC (2014). Probing emotional influences on cognitive control: an ALE meta-analysis of cognition emotion interactions. Brain Struct Funct.

[14] Schweizer S, Satpute AB, Atzil S, Field AP, Hitchcock C, Black M (2019). The impact of affective information on working memory: a pair of meta-analytic reviews of behavioral and neuroimaging evidence. Psychol Bull.

[15] Joormann J, Levens SM, Gotlib IH (2011). Sticky thoughts: depression and rumination are associated with difficulties manipulating emotional material in working memory. Psychol Sci.

[16] Yoon KL, Lemoult J, Joormann J (2014). Updating emotional content in working memory: a depression-specific deficit?. J Behav Ther Exp Psychiatry.

[17] Joormann J, Gotlib IH (2008). Updating the Contents of working memory in depression: interference from irrelevant negative material. J Abnorm Psychol.

[18] Levens SM, Gotlib IH (2010). Updating positive and negative stimuli in working memory in depression. J Exp Psychol.

[19] Wante L, Mueller SC, Cromheeke S, Braet C (2018). The impact of happy and angry faces on working memory in depressed adolescents. J Exp Child Psychol.

[20] Ladouceur CD, Dahl RE, Williamson DE, Birmaher B, Ryan ND, Casey BJ (2005). Altered emotional processing in pediatric anxiety, depression, and comorbid anxiety-depression. J Abnorm Child Psychol.

[21] Tavitian LR, Ladouceur CD, Nahas Z, Khater B, Brent DA, Maalouf FT (2014). Neutral face distractors differentiate performance between depressed and healthy adolescents during an emotional working memory task. Eur Child Adolesc Psychiatry.

[22] Strauss MM, Makris N, Aharon I, Vangel MG, Goodman J, Kennedy DN (2005). fMRI of sensitization to angry faces. Neuroimage.

[23] Luna B, Garver KE, Urban TA, Lazar NA, Sweeney JA (2004). Maturation of cognitive processes from late childhood to adulthood. Child Dev.

[24] Cromheeke S, Mueller SC (2016). The power of a smile: Stronger working memory effects for happy faces in adolescents compared to adults. Cogn Emot.

[25] Hardin MG, Schroth E, Pine DS, Ernst M (2007). Incentive-related modulation of cognitive control in healthy, anxious, and depressed adolescents: Development and psychopathology related differences. J Child Psychol Psychiatry Allied Discip.

[26] Schweizer S, Parker J, Leung JT, Griffin C, Blakemore SJ (2019). Age-related differences in affective control and its association with mental health difficulties. Dev Psychopathol.

[27] Mueller SC, Cromheeke S, Siugzdaite R, Nicolas Boehler C (2017). Evidence for the triadic model of adolescent brain development: cognitive load and task-relevance of emotion differentially affect adolescents and adults. Dev Cogn Neurosci.

[28] Casey BJ, Jones RM, Levita L, Libby V, Pattwell SS, Ruberry EJ (2010). The storm and stress of adolescence: insights from human imaging and mouse genetics. Dev Psychobiol.

[29] Deng X, Sang B, Ku Y, Sai L (2019). Age-related differences in the late positive potential during emotion regulation between adolescents and adults. Sci Rep.

[30] Kovacs M (1992). The children’s depression inventory: manual.

[31] Beck AT, Steer RA, Brown GK (1996). Beck depression inventory-II.

[32] Rønning JA, Haavisto A, Nikolakaros G, Helenius H, Tamminen T, Moilanen I (2011). Factors associated with reported childhood depressive symptoms at age 8 and later self-reported depressive symptoms among boys at age 18. Soc Psychiatry Psychiatr Epidemiol.

[33] Viinamäki H, Tanskanen A, Honkalampi K, Koivumaa-Honkanen H, Haatainen K, Kaustio O (2004). Is the beck depression inventory suitable for screening major depression in different phases of the disease?. Nord J Psychiatry.

[34] Erford BT, Johnson E, Bardoshi G (2016). Meta-Analysis of the English Version of the Beck Depression Inventory–Second Edition. Meas Eval Couns Dev.

[35] Li L, Lok GKI, Mei S-L, Cui X-L, An F-R, Li L (2020). Prevalence of depression and its relationship with quality of life among university students in Macau, Hong Kong and mainland China. Sci Rep.

[36] Selçuk EB, Demir AÇ, Erbay LG, Özcan ÖÖ, Gürer H, Dönmez YE (2021). Anxiety, depression and post-traumatic stress disorder symptoms in adolescents during the COVID‐19 outbreak and associated factors. Int J Clin Pract.

[37] Tottenham N, Tanaka JW, Leon AC, McCarry T, Nurse M, Hare TA (2009). The NimStim set of facial expressions: Judgments from untrained research participants. Psychiatry Res.

[38] Langner O, Dotsch R, Bijlstra G, Wigboldus DHJ, Hawk ST, van Knippenberg A (2010). Presentation and validation of the Radboud Faces Database. Cogn Emot.

[39] American Psychiatric Association. Diagnostic and statistical manual of mental disorders. 4th edition. Washington, DC; 2000.

[40] Roelofs J, van Breukelen G, de Graaf LE, Beck AT, Arntz A, Huibers MJH (2013). Norms for the Beck Depression Inventory (BDI-II) in a Large Dutch Community Sample. J Psychopathol Behav Assess.

[41] Timbremont B, Braet C. Children’s Depression Inventory, Nederlandstalige versie. 2002.

[42] Spielberger CD, Gorsuch RL, Lushene R, Vagg PR, Jacobs GA (1983). Manual for the state-trait anxiety inventory.

[43] Bakker FC, van Wieringen PCW, van der Ploeg H, Spielberger CD. Handleiding bij de Zelf-Beoordelings-Vragenlijst voor kinderen (ZBV-K). Swets & Zeitlinger; 1989.

[44] Spielberger CD, Edwards CD, Lushene RE, Montuori J, Platzek D (1973). State-trait anxiety inventory for children.

[45] Van der Ploeg H. Handleiding bij de Zelf-Beoordelings Vragenlijst. 2nd edition. Lisse: Swets Test Publishers; 2000.

[46] Kessler RC, Sampson NA, Berglund P, Gruber MJ, Al-Hamzawi A, Andrade L (2015). Anxious and non-anxious major depressive disorder in the World Health Organization World Mental Health Surveys. Epidemiol Psychiatr Sci.

[47] McTeague LM, Huemer J, Carreon DM, Jiang Y, Eickhoff SB, Etkin A (2017). Identification of common neural circuit disruptions in cognitive control across psychiatric disorders. Am J Psychiatry.

[48] Moran TP (2016). Anxiety and working memory capacity: a meta-analysis and narrative review. Psychol Bull.

[49] Rottenberg J, Gross JJ, Gotlib IH (2005). Emotion context insensitivity in major depressive disorder. J Abnorm Psychol.

[50] Hardin MG, Mandell D, Mueller SC, Dahl RE, Pine DS, Ernst M (2009). Inhibitory control in anxious and healthy adolescents is modulated by incentive and incidental affective stimuli. J Child Psychol Psychiatry.

[51] Pessoa L, Padmala S, Morland T (2005). Fate of unattended fearful faces in the amygdala is determined by both attentional resources and cognitive modulation. Neuroimage.

[52] Pessoa L. Cognitive control and emotional processing. Wiley Handb Cogn Control. 2017;:392–407.

[53] Lavie N, Hirst A, de Fockert JW, Viding E (2004). Load theory of selective attention and cognitive control. J Exp Psychol.

[54] Rock PL, Roiser JP, Riedel WJ, Blackwell AD (2014). Cognitive impairment in depression: a systematic review and meta-analysis. Psychol Med.

[55] Wagner S, Müller C, Helmreich I, Huss M, Tadić A (2015). A meta-analysis of cognitive functions in children and adolescents with major depressive disorder. Eur Child Adolesc Psychiatry.

[56] Schweizer S, Gotlib IH, Blakemore SJ (2020). The role of affective control in emotion regulation during adolescence. Emotion.

[57] Becker DV, Anderson US, Mortensen CR, Neufeld SL, Neel R (2011). The face in the crowd effect unconfounded: happy faces, not angry faces, are more efficiently detected in single- and multiple-target visual search tasks. J Exp Psychol Gen.

[58] Juth P, Lundqvist D, Karlsson A, Öhman A (2005). Looking for foes and friends: perceptual and emotional factors when finding a face in the crowd. Emotion.

[59] Figueira JSB, Pacheco LB, Lobo I, Volchan E, Pereira MG, de Oliveira L, et al. “Keep That in Mind!” The role of positive affect in working memory for maintaining goal-relevant information. Front Psychol. 2018;9. 10.3389/fpsyg.2018.01228. 10.3389/fpsyg.2018.01228 PMC6060567 30072937

[60] Bourke C, Douglas K, Porter R (2010). Processing of facial emotion expression in major depression: a review. Aust N Z J Psychiatry.

[61] Lu S, Xu J, Li M, Xue J, Lu X, Feng L (2017). Attentional bias scores in patients with depression and effects of age: a controlled, eye-tracking study. J Int Med Res.

[62] Duque A, Vázquez C (2015). Double attention bias for positive and negative emotional faces in clinical depression: evidence from an eye-tracking study. J Behav Ther Exp Psychiatry.

[63] Gotlib IH, Kasch KL, Traill S, Joormann J, Arnow BA, Johnson SL (2004). Coherence and specificity of information-processing biases in depression and social phobia. J Abnorm Psychol.

[64] Hankin BL, Gibb BE, Abela JRZ, Flory K (2010). Selective attention to affective stimuli and clinical depression among youths: role of anxiety and specificity of emotion. J Abnorm Psychol.

[65] Xu Q, Ye C, Gu S, Hu Z, Lei Y, Li X (2021). Negative and positive bias for emotional faces: evidence from the attention and working memory paradigms. Neural Plast.

[66] Harvey PO, Fossati P, Pochon JB, Levy R, LeBastard G, Lehéricy S (2005). Cognitive control and brain resources in major depression: an fMRI study using the n-back task. Neuroimage.

[67] Barkus E (2020). Effects of working memory training on emotion regulation: transdiagnostic review. PsyCh J.

[68] Beloe P, Derakshan N (2020). Adaptive working memory training can reduce anxiety and depression vulnerability in adolescents. Dev Sci.

[69] Lindsay EK, Chin B, Greco CM, Young S, Brown KW, Wright AGC (2018). How mindfulness training promotes positive emotions: dismantling acceptance skills training in two randomized controlled trials. J Pers Soc Psychol.

[70] Sanger KL, Thierry G, Dorjee D (2018). Effects of school-based mindfulness training on emotion processing and well-being in adolescents: evidence from event-related potentials. Dev Sci.

[71] Quach D, Jastrowski Mano KE, Alexander K (2016). A randomized controlled trial examining the effect of mindfulness meditation on working memory capacity in adolescents. J Adolesc Heal.

