# Supplementary material for: Comparing emotional working memory in adolescents and young adults with and without depressive symptoms: developmental and psychopathological differences
Source: BMC Psychol. 2022 May 25;10:134. doi: 10.1186/s40359-022-00836-2 (PMC9131646; doi:10.1186/s40359-022-00836-2)
Supplement: Supplementary file 1 — Additional file 1. Supplementary Table 1. Mean RTs (in Milliseconds) and Accuracy Rates (in %) on the 0-Back Task and the 2-Back Task for Young Adults and Adolescents as a Function of Psychopathology Group. [file 40359_2022_836_MOESM1_ESM.docx]

**Supplementary material**

**Reaction Time**

**Significant lower order interactions in the 5-way interaction**

There was a significant main effect of: load (F(1, 155) = 418.26 p < .001, ηp² = .73) with slower RTs for the 2-back condition; and emotion (F(21, 310) = 21.00, p < .001, ηp² = .12) with faster RTs for happy emotions compared to neutral (t(165) = -6.44, p < .001, d = .50) and angry (t(165) = 6.83, p < .001, d = .53). There was also a main effect of age group (F(1, 155) = 9.26, p = .003, ηp² = .06) adolescents showed slower RTs; and anxiety (F(1, 155) = 3.93, p = .049, ηp² = .03).

Other significant interactions emerged: load by emotion (F (2, 310) = 4.06, p = .018, ηp² = .03), task by emotion (F(2, 310) = 9.50, p < .001, ηp² = .06), load, task by emotion (F(2, 310) = 7.16, p = .001, ηp² = .04).

**Significant lower order interactions in the 4-way interaction in the adolescent group**

There as 3-way interaction between load, task and emotion (F(2, 130) = 3.44, p = .035, ηp² = .05). There was also an interaction between task and emotion, (F(2, 130) = 4.21, p =.017, ηp² = .06). There was a main effect of load (F(1, 65) = 130.19, p < .001, ηp² = .67), indicating slower RTs for high load; emotion (F(2, 130) = 5.47, p = .005, ηp² = .08), indicating faster RTs for happy compared to anger (t(73) = 3.46, p < .001, d = .40) and neutral (t(73) = -3.61, p < .001, d = .42) faces.; and psychopathology group (F(1, 65) = 5.15, p = .027, ηp² = .07) with slower RTs for adolescents in the depressive symptom group.

**Accuracy**

The load by emotion interaction (F(2, 310) = 3.44, p = .033, ηp² = .02) indicated lower error rates for neutral and happy faces relative to angry faces (t(163) = -6.33, p < .001, d = .50 and t(163) = -7.74, p < .001, d = .61, respectively). By contrast, in the high load condition accuracy was higher for happy emotions relative to both neutral (t(163) = 3.99, p < .001, d = .31) and angry faces (t(163) = -6.51, p < .001, d = .51). Accuracy was also higher for neutral than for anger (t(163) = -2.48, p = .045, d = 0.19). The load by task interaction (F(1, 155) = 6.00, p = .015, ηp² = .04) indicated a higher accuracy for valence (t(164) = 16.35, p < .001, d = 1.27) and gender (t(164) = 15.91, p < .001, d = 1.24) conditions in low load when compared to high load. Unsurprisingly, main effects of load, age group, and emotion also emerged showing: 1) higher accuracy during low vs. high load, (F(1, 155) = 341.23, p < .001, ηp² = .691); 2) better performance for young adults relative to adolescents (F(1, 155) = 39.23, p < .001, ηp² = .20); and 3) higher accuracy during performance of happy emotions vs. angry emotions (t(163) = -8.97, p < .001, d = .70); and higher on happy vs. neutral (t(163) = 3.10, p = .004, d = .24), as well as higher accuracy on neutral vs. angry emotions (t(163) = -6.07, p < .001, d =.48).

| **Supplementary Table 1**  Mean RTs (in Milliseconds) and Accuracy Rates (in %) on the 0-Back Task and the 2-Back Task for Young Adults and Adolescents as a Function of Psychopathology Group*.* | | | | | | | | | | | | | | |
| --- | --- | --- | --- | --- | --- | --- | --- | --- | --- | --- | --- | --- | --- | --- |
|  |  | Young Adults | | | | | |  | Adolescents | | | | | |
|  |  | Total | | Depressive Symptoms | | Healthy | |  | Total | | Depressive Symptoms | | Healthy | |
| Condition | Emotion | RT | Acc | RT | Acc | RT | Acc |  | RT | Acc | RT | Acc | RT | Acc |
| **0-Back** |  |  |  |  |  |  |  |  |  |  |  |  |  |  |
| Gender | Angry | 750(18) | 89(1) | 737(25) | 89(1) | 758(24) | 89(2) |  | 829(17) | 87(1) | 784(28) | 85(2) | 847(20) | 87(1) |
|  | Happy | 725(16) | 94(1) | 713(26) | 95(1) | 732(21) | 83(2) |  | 820(15) | 91(1) | 780(32) | 89(2) | 836(17) | 91(1) |
|  | Neutral | 727(15) | 92(1) | 725(26) | 93(1) | 728(18) | 92(2) |  | 824(16) | 92(1) | 775(31) | 91(2) | 844(19) | 92(2) |
| Valence | Angry | 697(14) | 91(1) | 685(21) | 93(1) | 704(19) | 90(1) |  | 825(16) | 89(1) | 766(34) | 90(2) | 848(18) | 89(1) |
|  | Happy | 676(15) | 94(1) | 660(24) | 96(1) | 686(19) | 93(1) |  | 785(16) | 93(1) | 738(28) | 93(2) | 803(19) | 94(1) |
|  | Neutral | 738(16) | 95(1) | 729(26) | 96(1) | 743(20) | 94(1) |  | 861(18) | 92(1) | 813(34) | 92(2) | 880(21) | 92(1) |
| **2-Back** |  |  |  |  |  |  |  |  |  |  |  |  |  |  |
| Gender | Angry | 1059(21) | 77(1) | 1047(37) | 79(3) | 1066(26) | 76(2) |  | 1131(24) | 65(1) | 1050(41) | 63(3) | 1164(28) | 67(1) |
|  | Happy | 1030(22) | 80(1) | 1017(39) | 79(3) | 1039(26) | 80(1) |  | 1138(27) | 69(1) | 1010(49) | 68(2) | 1189(30) | 69(1) |
|  | Neutral | 1054(22) | 79(1) | 1025(39) | 79(3) | 1073(27) | 79(1) |  | 1111(25) | 69(1) | 1035(47) | 68(4) | 1141(28) | 69(2) |
| Valence | Angry | 1058(22) | 76(1) | 1049(37) | 77(2) | 1063(27) | 75(2) |  | 1126(24) | 66(1) | 1005(43) | 67(3) | 1175(27) | 66(2) |
|  | Happy | 1017(23) | 81(1) | 1011(37) | 80(2) | 1022(30) | 82(2) |  | 1097(24) | 70(2) | 1022(50) | 67(4) | 1127(27) | 71(2) |
|  | Neutral | 1042(21) | 77(1) | 1032(38) | 77(2) | 1048(25) | 77(2) |  | 1127(29) | 68(2) | 998(50) | 69(4) | 1178(33) | 67(2) |
| *Note*. Standard errors are in parentheses. Acc = Accuracy; RT = Reaction time. | | | | | | | | | | | | | | |
